# Synthesis and Application of Cobalt Oxide (Co_3_O_4_)-Impregnated Olive Stones Biochar for the Removal of Rifampicin and Tigecycline: Multivariate Controlled Performance

**DOI:** 10.3390/nano12030379

**Published:** 2022-01-24

**Authors:** Ahmed S. El-Shafie, Insharah Ahsan, Mohamed Radhwani, Mohammed Ali Al-Khangi, Marwa El-Azazy

**Affiliations:** 1 Department of Chemistry and Earth Sciences, College of Arts and Sciences, Qatar University, Doha P.O. Box 2713, Qatar; aelshafie@qu.edu.qa (A.S.E.-S.); ia1803723@student.qu.edu.qa (I.A.); 2 Al Jazeera Academy, Doha P.O. Box 22250, Qatar; 090055@aja.edu.qa (M.R.); asqtr74@gmail.com (M.A.A.-K.)

**Keywords:** cobalt oxide nanoparticles (Co_3_O_4_), olive stones biochar, rifampicin, tigecycline, wastewater, full factorial design

## Abstract

Cobalt oxide (Co_3_O_4_) nanoparticles supported on olive stone biochar (OSBC) was used as an efficient sorbent for rifampicin (RIFM) and tigecycline (TIGC) from wastewater. Thermal stabilities, morphologies, textures, and surface functionalities of two adsorbents; OSBC and Co-OSBC were compared. BET analysis indicated that Co-OSBC possesses a larger surface area (39.85 m^2^/g) and higher pore-volume compared to the pristine OSBC. FT-IR analysis showed the presence of critical functional groups on the surface of both adsorbents. SEM and EDX analyses showed the presence of both meso- and macropores and confirmed the presence of Co_3_O_4_ nanoparticles on the adsorbent surface. Batch adsorption studies were controlled using a two-level full-factorial design (2*^k^*-FFD). Adsorption efficiency of Co-OSBC was evaluated in terms of the % removal (%R) and the sorption capacity (*q_e_*, mg/g) as a function of four variables: pH, adsorbent dose (AD), drug concentration, and contact time (CT). A %R of 95.18% and 75.48% could be achieved for RIFM and TIGC, respectively. Equilibrium studies revealed that Langmuir model perfectly fit the adsorption of RIFM compared to Freundlich model for TIGC. Maximum adsorption capacity (*q_max_*) for RIFM and TIGC was 61.10 and 25.94 mg/g, respectively. Adsorption kinetics of both drugs could be best represented using the pseudo-second order (PSO) model.

## 1. Introduction

Pharmaceutically active chemicals (PhACs), an important group of emergent contaminants, have attracted a lot of attention since they were discovered in surface and wastewater treatment plants in the late 1990s. Approximately 3000 different types of PhACs are routinely employed in the different therapeutic rehearsals, and more than 80 species have been detected in diverse environmental matrices across the world. It is expected that worldwide consumption of PhACs sums to some 100,000 tons or more per year. Therefore, the existence and fate of PhACs has become a major concern and a focus of research for the 21st century [1,2,3,4].

**Scheme 1:**
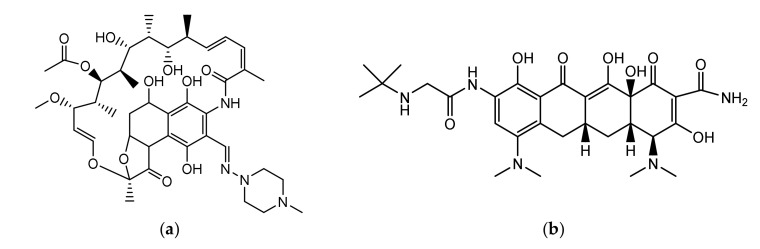
Chemical structure of: (**a**) Rifampicin (RIFM): C_43_H_58_N_4_O_12_ (822.94 g/mol), pK_a1_ = 1.7 and pK_a2_ = 7.9 [14]; (**b**) Tigecycline (TIGC): (585.65 g/mol), pKa values: 2.8, 4.4, 7.4, 8.9, and 9.5 [15,16].

Antibiotics represent a major class of antimicrobial agents that are commonly used for therapeutic purposes. Literature surveying shows the wide consumption of antibiotics. In 2002, the world-wide consumption of antibiotics ranged between 100,000–200,000 tons. The global consumption of antibiotics per capita has increased by 39% between 2000–2015 [5,6,7]. Veterinary antibiotics are also online and their removal is even more challenging [8]. One of the major concerns related to the contamination of the aquatic environments by antibiotics in specific is the potential for such agents to stimulate the selection and development of antimicrobial drug resistance among the microorganisms, flora and fauna living in the polluted sites. Rifampicin (RIFM) and tigecycline (TIGC), Scheme 1, are among the antibiotics with reported drug resilience [9,10,11,12,13].

RIFM is a semisynthetic antimicrobial with a broad spectrum of activity and is commonly used to treat tuberculosis (TB), and meningitis [11,17,18]. Recent investigations showed the potential of RIFM as a repurposed drug for the treatment of COVID-19 [19]. RIFM can cause gastrointestinal distress and hepatotoxicity. The current treatment technologies are incapable of totally removing RIFM. The leakage of RIFM into the water matrices during the manufacturing or application processes is likely to develop antimicrobial resistance, and persistent toxicity [17,20]. TIGC, a member of the tetracyclines family, is used to treat infections caused by multidrug-resistant bacteria [9,21]. TIGC is mostly eliminated unaltered in feces (59%) and urine (22%) [22]. As of 2010 and following the appearance of cases of unknown deaths associated with TIGC administration, the FDA has issued a black box warning for the drug [23]. Removal of these antibiotics is therefore a must.

The growing demand for low-cost, high-performance, and easy-to-handle materials for wastewater cleanup has prompted the scientific community to look into the valorization of agro-wastes into value-added products [24]. Biochar (BC)—an advanced carbon-based material—is the carbonaceous commodity produced via pyrolysis of biomasses at high temperatures and in an oxygen-free environment [25]. The fascinating physicochemical characteristics of the BC (high surface area, porosity, possibility of functionalization and tailoring for specific contaminant removal) laid back with a possibility for production at both laboratory and industrial scales make it a promising and sustainable remedy for wastewater treatment. A unique feature of BC is the surface structure which is controlled mainly by micropores-that facilitate removal of small molecules- followed by mesopores and macropores. The presence of meso- and macropores enables the diffusion of antibiotics into the adsorbent with faster transfer rates [4,26,27,28,29,30,31,32]. Efforts to generate engineered BC which retains well-customized pore dimensions, and wider structural heterogeneity have been excreted. One of the effective approaches to stimulate meso- and macropore formation is activation via transition metals which act by pitting holes in the carbonaceous matrix [33,34].

Biochar of olive stones (OSBC) either pristine or engineered has recently garnered the attention as an adsorbent for removing aquatic contaminants [35,36,37]. Conversion of olive stones into BC-based adsorbent is a sustainable solution for the disposal problems confronted by the well-established olive oil industry, where the total waste generation is nearly 75% of the olive harvest [38]. In the current approach, a microemulsion-assisted synthesis of cobalt oxide nanoparticles–loaded OSBC (Co-OSBC) adsorbent will be executed using oleylamine as a surfactant. The objective of following such an approach is to increase the surface area and generate more active sites on the surface of the OSBC, where existence of cobalt oxide (Co_3_O_4_) nanoparticles could serve as extra binding sites that facilitate antibiotic trapping and increase the surface area. Presence of the surfactant, oleylamine, facilitates the formation of small and uniform-sized nanoparticles.

Moreover, and with an objective to develop an eco-structured nanosorbent with preservation of the process greenness, the performance of the developed nanosorbent in depolluting RIFM and TIGC will be controlled employing a multivariate approach—two-level full factorial design (2*^k^*-FFD, where *k* is the number of variables). Variables affecting the adsorption efficiency of the Co-OSBC (pH, dose of Co-OSBC, contact time (CT), and concentration of the pollutant) will be studied. Each independent variable will be studied at two levels and two responses will be assessed: %R and *q*_e_ [39,40]. Factorial-based experiments provide several advantages, including fewer trials and hence saving of resources with less waste generation, a better opportunity to investigate variable-variable interactions, and output data that could be treated with high degree of inevitability. Few efforts are reflected in the literature on using BC-based nanosorbents for antibiotics removal with a multivariate-controlled performance. Table 1 shows some of the reported investigations. The impact of loading the BC with nanoparticles on performance of the obtained nanosorbents is revealed in terms of surface area, % removal (%R) and adsorption capacity (*q*_e_, mg/g). Table 1 also shows the approaches followed for optimization of process variables, where most of the reported approaches were univariate-based [41,42,43,44,45,46,47].

The uniqueness of the current approach and to the best of our knowledge is, therefore, being the first report on the removal of two important antibiotics in a single mode system, RIFM and TIGC, using a transition metal decorated-BC obtained via pyrolysis of an abundant agro-waste with the performance being tuned using a multivariate scheme.

## 2. Materials and Methods

### 2.1. Materials, Equipment, and Software

Chemicals used in this study including sodium hydroxide, hydrochloric acid, sodium chloride, oleylamine, n-propanol, and cobalt (II) chloride hexahydrate (CoCl_2_·6H_2_O) were purchased from Sigma–Aldrich (St. Louis, MO, USA). Rifampicin (RIFM) and tigecycline (TIGC) and were purchased from Biosynth^®^ Carbosynth Ltd. (Compton, Berkshire, UK). Deionized water used throughout this study was prepared using Millipore-Q water system (Burlington, MA, USA). Olives were purchased from local markets in Doha–Qatar. To dry the olive stones, an oven (Memmert, GmbH + Co. KG, Schwabach, Germany) was utilized. Stones were burnt in the furnace (Barnstead, Dubuque, IA, USA). A stock solution of 200 ppm of both RIFM and TIGC was prepared by dissolving the prerequisite drug amounts into deionized water and then sonicating the solution for 30 min till complete dissolving using Bransonic^®^ sonicator (Ultrasonic bath, Emerson, CT, USA). The solution pH in which the adsorbents were suspended was adjusted using either 0.1 M NaOH or 0.1 M HCl. Vernier LabQuest pH meter was used for the pH measurements. Concentrations of RIFM and TIGC before and after adsorption were measured using a UV-Vis spectrophotometer (Agilent diode-array, Agilent, Santa Clara, CA, USA) with 10 mm matched quartz cuvettes. The reaction mixture was filtered using a Millex syringe filter (nylon, non-sterile, 0.45 µm).

Fourier transform infrared spectroscopy (FT-IR, Perkin Elmer, Shelton, CT, USA) was utilized to explore the functional groups on the adsorbent surface. Scanning electron microscopy (SEM, FEI, Quanta 200, Thermo Scientific, Waltham, MA, USA) was used to examine the surface morphology of the studied sorbent. Energy-dispersive X-ray spectrometer (EDX) was utilized to determine the elemental composition on the Co-OSBC surface. Further elemental analysis and investigation of the carbonaceous nature for Co-OSBC was carried out using Raman spectroscopy (Thermo Scientific, Waltham, MA, USA). The microstructural characterization of Co-OSBC was investigated using a transmission electron microscope (TEM, FEI, TECNAI G2 TEM, TF20, FEI, Hillsboro, OR, USA). Micrometrics ASAP2020 accelerated surface area, the porosimetry system, (Micrometrics, Norcross, GA, USA) was used to analyze the surface characteristics such as pore size, surface area, and pore volume, and it was conducted by degassing Co-OSBC sample followed by the N_2_ adsorption-desorption study. The isotherms measured at 77 K were used along with applying the Brunauer–Emmett–Teller (BET) equation to calculate the surface area. On the other hand, the t-plots were used with Barrett–Joyner–Halenda (BJH) equations to estimate the pore volume.

Minitab^®^19 software was obtained from Minitab Inc. (State College, PA, USA) and it was utilized to structure and analyze the FFD design.

### 2.2. Preparation of Olive Stone Biochar (OSBC)

Olive stones were taken out from the olives, washed 10 times using tap water, followed by washing other 10 times using deionized water. The clean stones were then placed in the oven at 80 °C for three days, and then portions of the olive stones were crushed and sieved using a 0.125 mm sieve. The stones powder was placed in crucibles and burnt in the furnace at 500 °C for 1 h. The produced BC was further grinded and sieved using a 0.125 mm sieve. Finally, the obtained powder (OSBC) was placed in a sealed bottle and kept for further use.

### 2.3. Synthesis of Co-Olive Stone Biochar (Co-OSBC)

Co_3_O_4_ nanoparticles supported on OSBC were synthesized using a microemulsion–based method with minor modifications in the surfactant type [48]. The Co-OSBC was prepared by dissolving 4.9327 g of CoCl_2_·6H_2_O, which equivalent to 1:10 (Co:OSBC) (*w/w*) in 200 mL of deionized water followed by the addition of 10 g of the OSBC with a continuous stirring using fixed stirring speed of 800 rpm. The second step is the addition of 100 mL of 0.1 M oleylamine dissolved in n-propanol into the reaction mixture with stirring for 3 h at room temperature. The precipitation of the cobalt nanoparticles was achieved by adding droplets of ammonia solution (26%) until pH 12. Finally, the product (Co-OSBC) was separated from the solution using a centrifuge at 4000 rpm speed for 10 min. The product was washed 5 times using deionized water followed by ethanol and then dried in the oven at 70 °C for 24 h. The resulting product Co-OSBC was then characterized using TGA, FT-IR, Raman, BET, TEM, SEM, and EDX analyses.

### 2.4. Point-of-Zero-Charge (pH_PZC_)

Determination of the pH_PZC_ for both samples OSBC and Co-OSBC by mixing an equal amount (~1.0 ± 0.005 g) of the two samples separately into seven flasks and each flask containing 50 mL of NaCl solution (0.01 M). The studied pH in each flask was adjusted between 3.0–9.0 ± 0.2 using either 0.1 M HCl or 0.1 M NaOH. Samples were mixed and stirred for 48 h in the automatic shaker at 150 rpm followed by measuring the final pH. The pH_PZC_ was then determined from the intersection point of the curve between pH_final_ versus the pH_initial_ [49].

### 2.5. Investigation of Variables Affecting the Sorption Efficiency of Co-OSBC Using Full Factorial Design (FFD)

In the current study, a full factorial design (FFD) was used to optimize the performance of the Co-OSBC in depolluting RIFM and TIGC. Four main factors were tested, including pH, drug concentration [Antibiotic], dose of Co-OSBC (AD), and contact time (CT), Table 2 (lower bound is denoted as −1, while the upper bound is denoted as +1). The target was to maximize two responses, %R and *q_e_* (mg/g) as a function of the four independent variables. These two parameters were calculated using Equations (1) and (2). The design output involved 20 experimental runs encompassing 4 central points (Ct Pt, denoted as 0). The design was conducted over 4 blocks, Table 3.

(1) $$\left(\%R\right)=\frac{C_{0}-C_{e}}{C_{0}}\times 100\%$$

(2) $$\left(q_{e}\right)=\frac{C_{0}-C_{e}}{W}\;V$$

where C_0_ is the initial concentration of both drugs [TIGC] and [RIFM] solution in ppm, while C_e_ is the concentration of these two drug solutions at equilibrium in ppm, V is the volume of the drug solution (L), and W is the mass of the Co-OSBC adsorbent (g).

### 2.6. Equilibrium and Kinetic Studies

The equilibrium studies were carried out by preparing a series of dilutions in deionized water in the range between 5–400 ppm for both RIFM and TIGC, and the pH value was adjusted to pH 5.00 ± 0.20 using 0.1 M HCl. Equal masses of 0.100 ± 0.005 g of the studied adsorbent (Co-OSBC) were added to each concentration from both TIGC and RIFM solution. The mixture was then shaken using an automatic shaker for an equilibrium time of 24 h at 150 rpm. The prepared solutions were filtered, and the absorbance was measured at 475 nm for RIFM and 347 nm for TIGC solutions.

The sorption kinetics for both drugs (TIGC and RIFM) were performed by mixing 150 mL of each drug solution (100 ppm, pH 5.00 ± 0.20) with ~1.0 g of Co-OSBC with continuous stirring at 150 rpm. An aliquot of 10 mL of the mixture was withdrawn and filtered using a syringe filter at different time ranges over 90 min. Finally, the absorbance of the filtrate was measured at 475 nm and 347 nm for RIFM and TIGC, respectively.

## 3. Results and Discussion

### 3.1. Adsorbents’ Characterization and Surface Chemistry

#### 3.1.1. Thermogravimetric Analysis (TGA)

**Figure 1:**
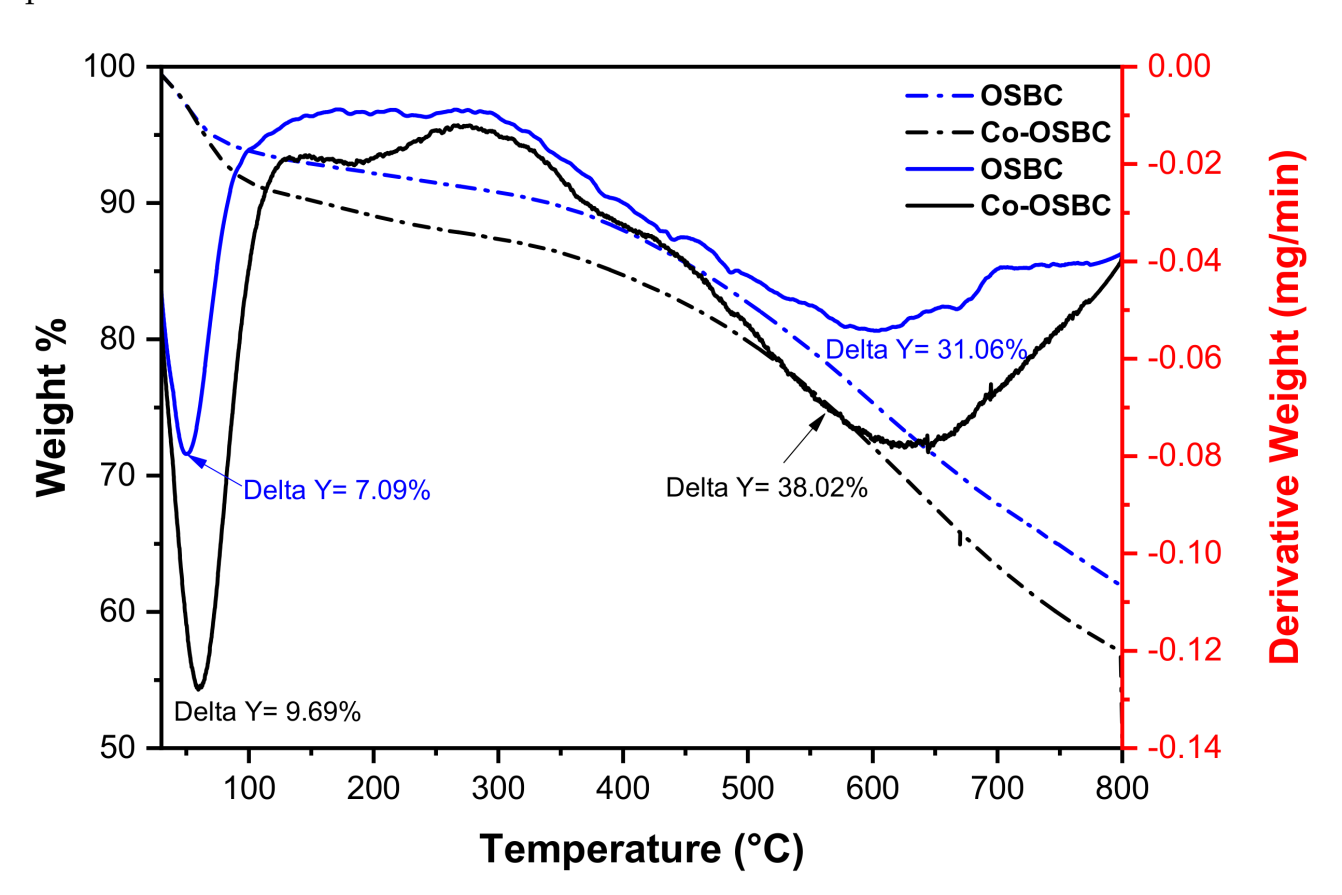
TGA/*d*TA analysis of OSBC and Co-OSBC.

The thermal stability of both OSBC and Co-OSBC was investigated using the TGA, Figure 1. The results reveal that both samples are thermally stable in the temperature range of 100–450 °C. The weight loss between 50 and 100 °C for OSBC and Co-OSBC was 7.09% and 9.69%, respectively, and may be ascribed to the free water vaporization. On the other hand, between 550–800 °C, a loss of 31.06% and 38.02% was observed for OSBC and Co-OSBC, respectively, which might be attributed to the loss of organic content or the carbonization of polymeric material.

#### 3.1.2. FT-IR Analysis and Point-of-Zero-Charge (pH_PZC_)

**Figure 2:**
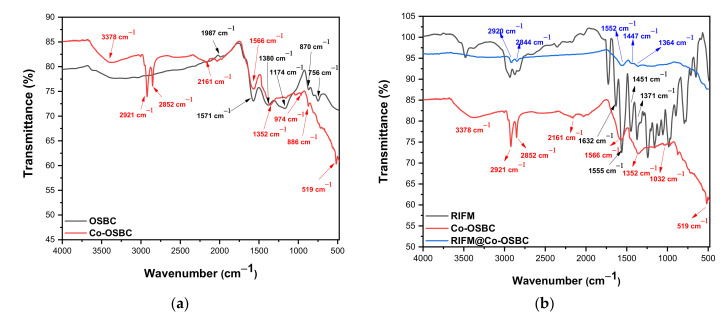

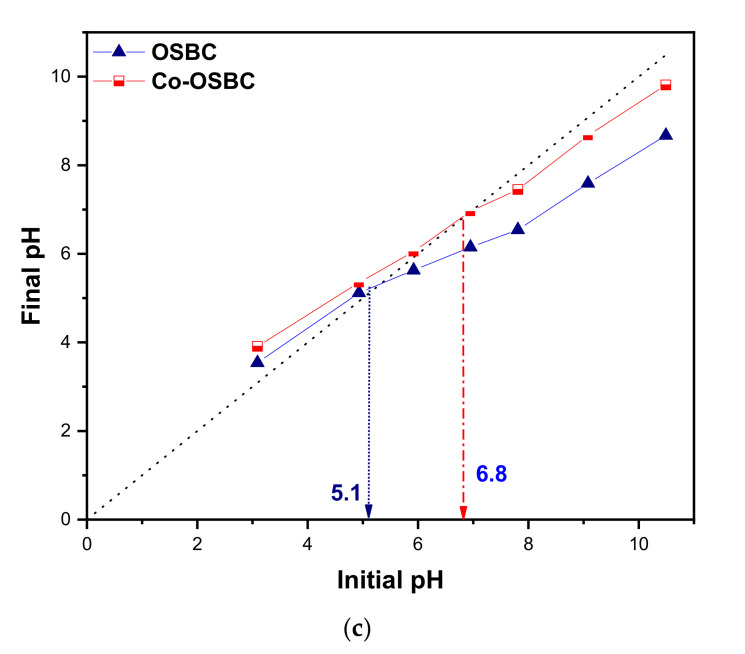
FT-IR spectra of OSBC and Co-OSBC (**a**) before adsorption, (**b**) free RIFM and Co-OSBC after adsorption, (**c**) pH_PZC_ for OSBC and Co-OSBC adsorbents.

FT-IR was used to identify the functional groups on the surface of the as-prepared sorbents; Co-OSBC and OSBC. Figure 2a depicts the FT-IR spectrums of OSBC and Co-OSBC prior to adsorption. The acquired data demonstrate that the spectra of both adsorbents are almost identical, except for three strong peaks at 2921, 2852, and 519 cm^−1^ in the spectrum of the Co-OSBC. The use of oleylamine and alcohol during the synthesis of Co_3_O_4_ nanoparticles has resulted in two absorption peaks at 2921 and 2852 cm^−1^, that could be attributable to the O–H stretching of alcohols or the N–H stretching of amines. The characteristic absorption peak and at 519 cm^−1^, on the other hand, could be associated to the presence of Co–O group, confirming the development of Co_3_O_4_ nanoparticles on the surface of the OSBC [34,50]. The FT-IR spectrum of the OSBC sample shows an absorption band at 1571 cm^−1^ which might be related to the aromatic skeletal vibration in lignin. Furthermore, the two bands at 1380 cm^−1^ and 1174 cm^−1^ are associated with the C–H deformation and the C–O–C vibration, respectively. The absorption band at 870 cm^−1^ could be related to the C–H deformation in cellulose, whereas the band at 756 cm^−1^ is associated with the aryl C–H or aryl C–O groups [51]. These peaks appeared in the Co-OSBC IR spectrum with a slight shift, e.g., the absorption band at 1571 cm^−1^ in the OSBC is also present in Co-OSBC at 1566 cm^−1^.

Figure 2b depicts the FT-IR spectrum of the RIFM, Co-OSBC before adsorption, and Co-OSBC after adsorption of RIFM. The obtained data reveal the presence of characteristic peaks of free RIFM, including the two bands at 2934 and 2867 cm^−1^ which are associated with the C–H and =C–H stretching. Furthermore, the strong absorption band at 1555 cm^−1^ may be assigned to the C=C stretching [52,53]. Nevertheless, the FT-IR spectrum of RIFM@Co-OSBC shows the presence of the RIFM peaks with a slight shift such as the absorption band at 1555 cm^−1^ in the RIFM spectrum also appears at 1552 cm^−1^ after the adsorption process confirming the successful adsorption of the RIFM onto the Co-OSBC.

The pH_PZC_ of the as-prepared samples, Figure 2c revealed that OSBC has a pH_PZC_ of 5.1 and changed to 6.8 following the impregnation with the Co_3_O_4_ nanoparticles. These values are comparable to the previously reported values for OSBC [37,54]. As a result, at a pH of 5.0 ± 0.2 (lower bound), the surface of the adsorbent could be neutral to positively charged, but at a pH of 9.0 ± 0.2 (upper bound), the surface will be negatively charged. On the other hand, RIFM and TIGC are ampholytic with pKa values of 1.7 and 7.9 in case of RIFM and 2.8, 4.4, 7.4, 8.9, and 9.5 in case of TIGC, Scheme 1. Therefore, RIFM can be found as a zwitterion at pH 5.0. Therefore, the electrostatic interaction in case of RIFM with both adsorbents within the investigated pH range might not be the best explanation for this adsorption mechanism. In case of TIGC, at pH 9.0 which is less than the highest pKa, the drug might be positively charged, while the adsorbent’s surface is negatively charged, an issue which might suggest the occurrence of chemisorption.

#### 3.1.3. Raman Spectroscopy

**Figure 3:**
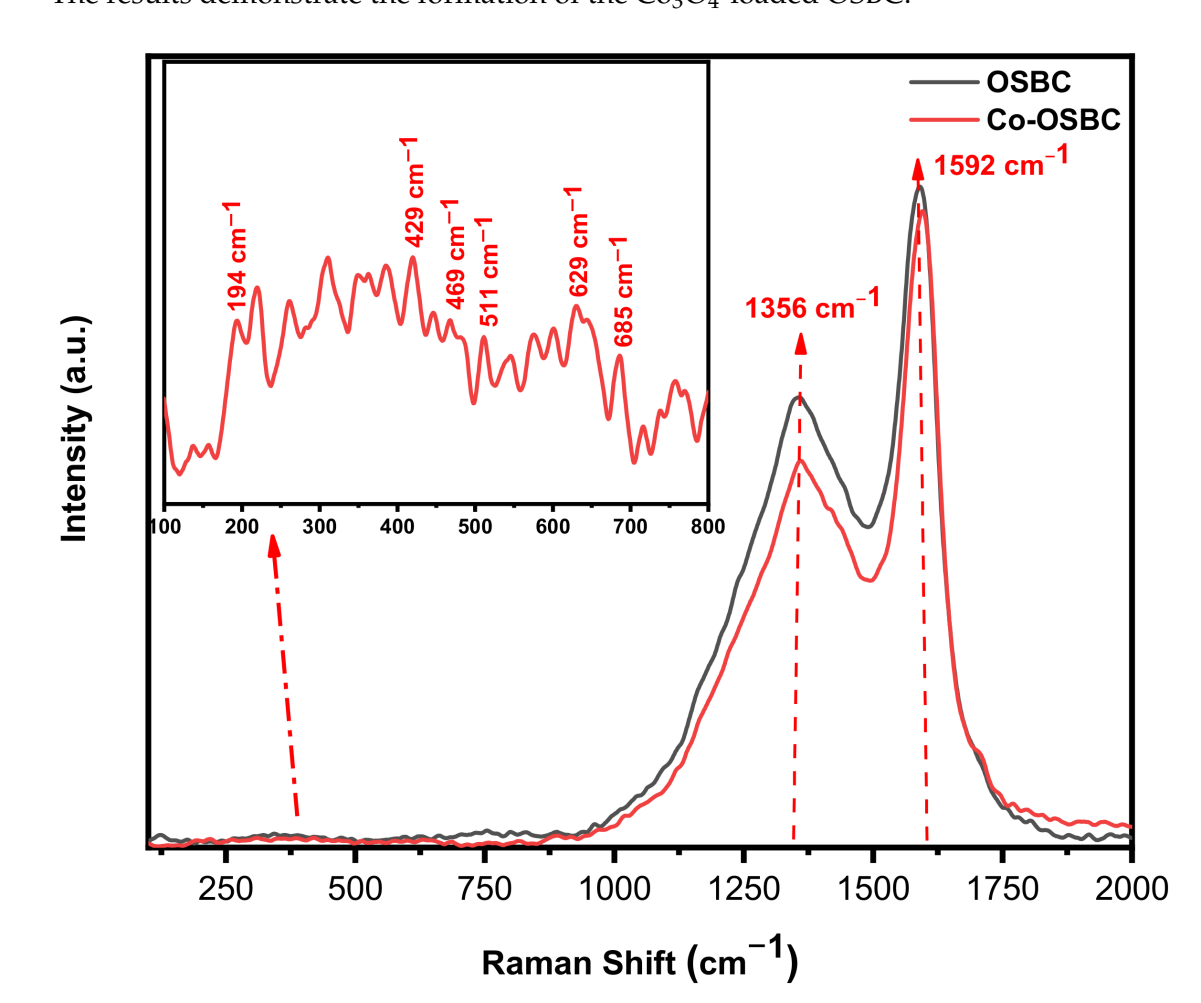
Raman spectra of the as-prepared sorbents OSBC and Co-OSBC. The inset is the Raman spectrum of Co-OSBC alone in the range of 100–800 cm^−1^.

Figure 3 depicts the Raman spectra of both adsorbents. Two separate bands, typical of carbonaceous materials, were found at 1356 cm^−1^ (D–band) and 1592 cm^−1^ (G–band). The D–band indicates carbon lattice features such as defects and sizes, whereas the G–band reflects C–C stretching for the *sp*^2^ system. In the case of OSBC, the intensity ratio of the two bands, *I_D_*:*I_G_*, was 0.67, compared to 0.60 in Co-OSBC. This observation demonstrates the presence of defects on the surface of OSBC, and it was reduced following loading with Co_3_O_4_ nanoparticles, which cover a portion of these defects. The spectra of Co-OSBC, on the other hand, exhibits five weak peaks centered at 685, 620, 511, 469, and 194 cm^−1^; these peaks indicate the Co_3_O_4_ spinel structure. The Raman mode at 685 cm^−1^ (A_1g_) is linked to octahedral site features, whereas the E_g_ (470 cm^−1^) and F_2g_ (194, 511, and 620 cm^−1^) modes are most likely associated to tetrahedral site and octahedral oxygen movements [55,56,57,58]. The results demonstrate the formation of the Co_3_O_4_-loaded OSBC.

#### 3.1.4. Textural Features

**Figure 4:**
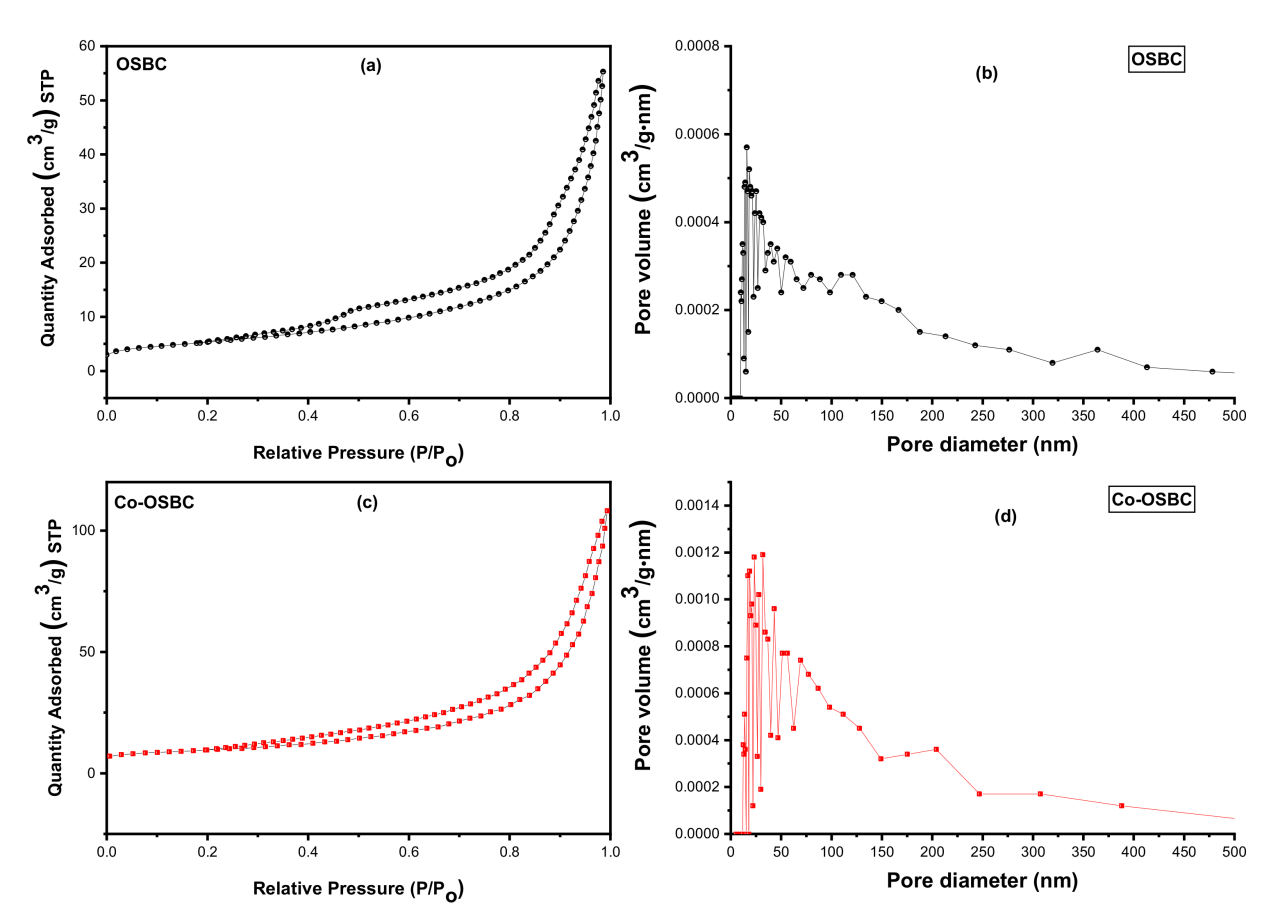
Nitrogen adsorption–desorption isotherms for (**a**) OSBC, (**c**) Co-OSBC and the pore diameter for (**b**) OSBC and (**d**) Co-OSBC.

Table 4 shows the surface area, pore volume, and pore radius determined using the BET equation for the as-prepared sorbents (OSBC and Co-OSBC). Figure 4 illustrates the N_2_ adsorption-desorption isotherms. The obtained data reveal that the Langmuir surface area rose from 22.20 m^2^/g in case of OSBC to 39.85 m^2^/g in case of Co-OSBC. This behavior might be related to the presence of Co_3_O_4_ nanoparticles on the surface of the OSBC, which increases surface area and hence improves removal performance towards RIFM and TIGC. In addition, both samples had two types of pores: mesopores (2–50 nm) and macropores (>50 nm). The adsorption isotherm was of type IV for both sorbents, signifying monolayer–multilayer adsorption followed by capillary condensation. In addition, the hysteresis loop was of the H3 type [59], which is typically seen on materials with a wide range of pore sizes, implying loose masses of plate-like particles producing slit-like pores.

#### 3.1.5. Morphological Features of OSBC and Co-OSBC: SEM, EDX, and TEM Analyses

**Figure 5:**
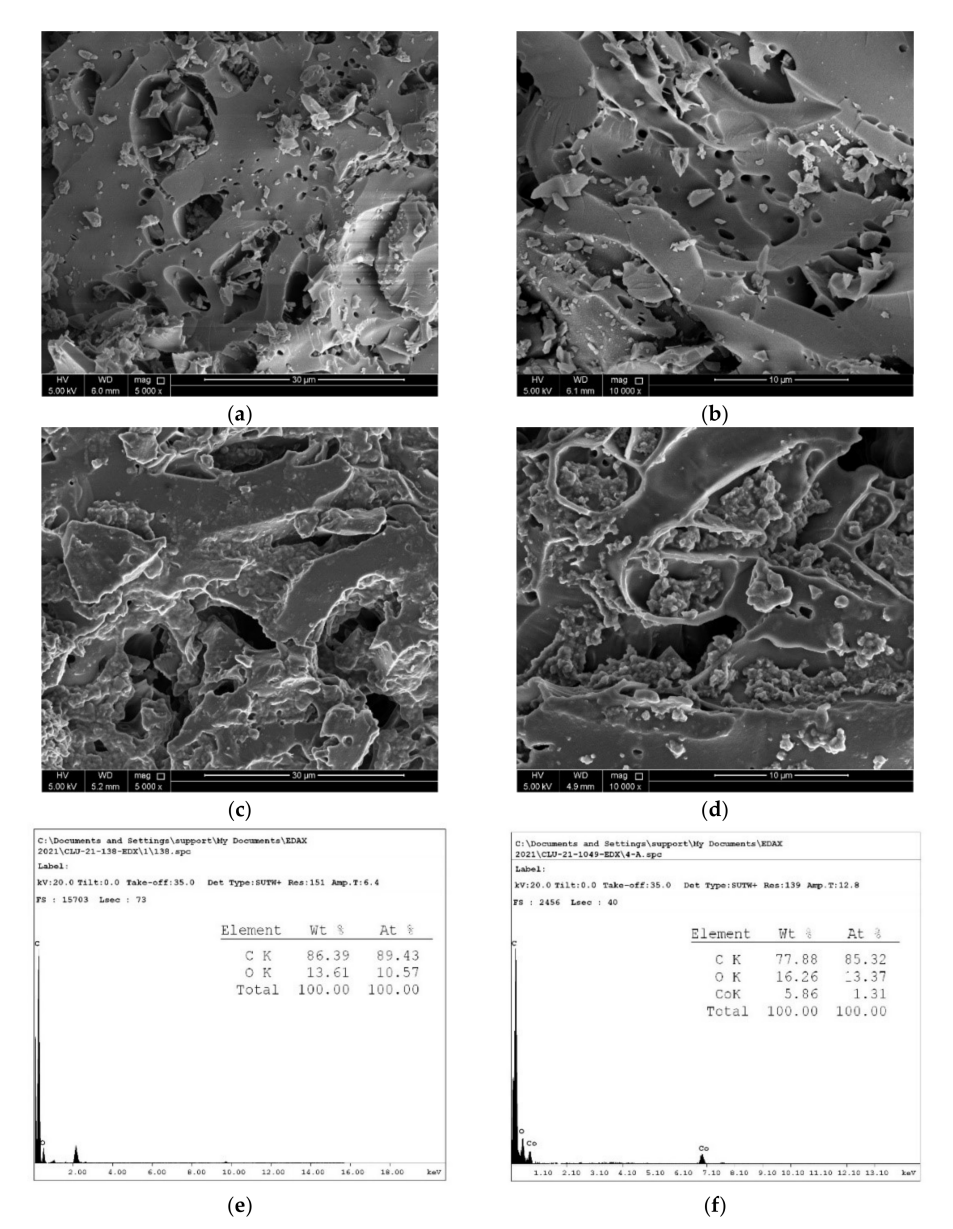
SEM micrographs of (**a**,**b**) OSBC, (**c**,**d**) Co-OSBC at 5000× and 10,000× magnifications, (**e**,**f**) EDX analyses of OSBC and Co-OSBC, respectively.

SEM, SEM–EDX, and TEM investigations were examine the surface morphology, macroporosity, and microscopic characteristics of OSBC and the prepared nanosorbent; Co-OSBC. Figure 5a,b show SEM micrographs of OSBC. As demonstrated by the BET analysis, the micrographs show the existence of several types of pores (mainly meso- and macropores) on the surface of the OSBC. In the SEM micrograph of Co-OSBC (Figure 5c,d), Co_3_O_4_ nanoparticles appear on the surface, confirming the formation of the Co-OSBC nanosorbent. The SEM findings were further supported by the EDX study shown in Figure 5e,f. The EDX analysis of the OSBC revealed a significant concentration of carbon (86.39%) and oxygen (13.61%), indicating the synthesis of carbonaceous material following thermal treatment of the olive stone biomass. EDX data for the Co-OSBC, on the other hand, indicates the presence of cobalt with a concentration of 5.86% and oxygen with a concentration of 16.26%, supporting the formation of Co_3_O_4_ nanoparticles on the surface of the olive stone biochar (OSBC).

**Figure 6:**
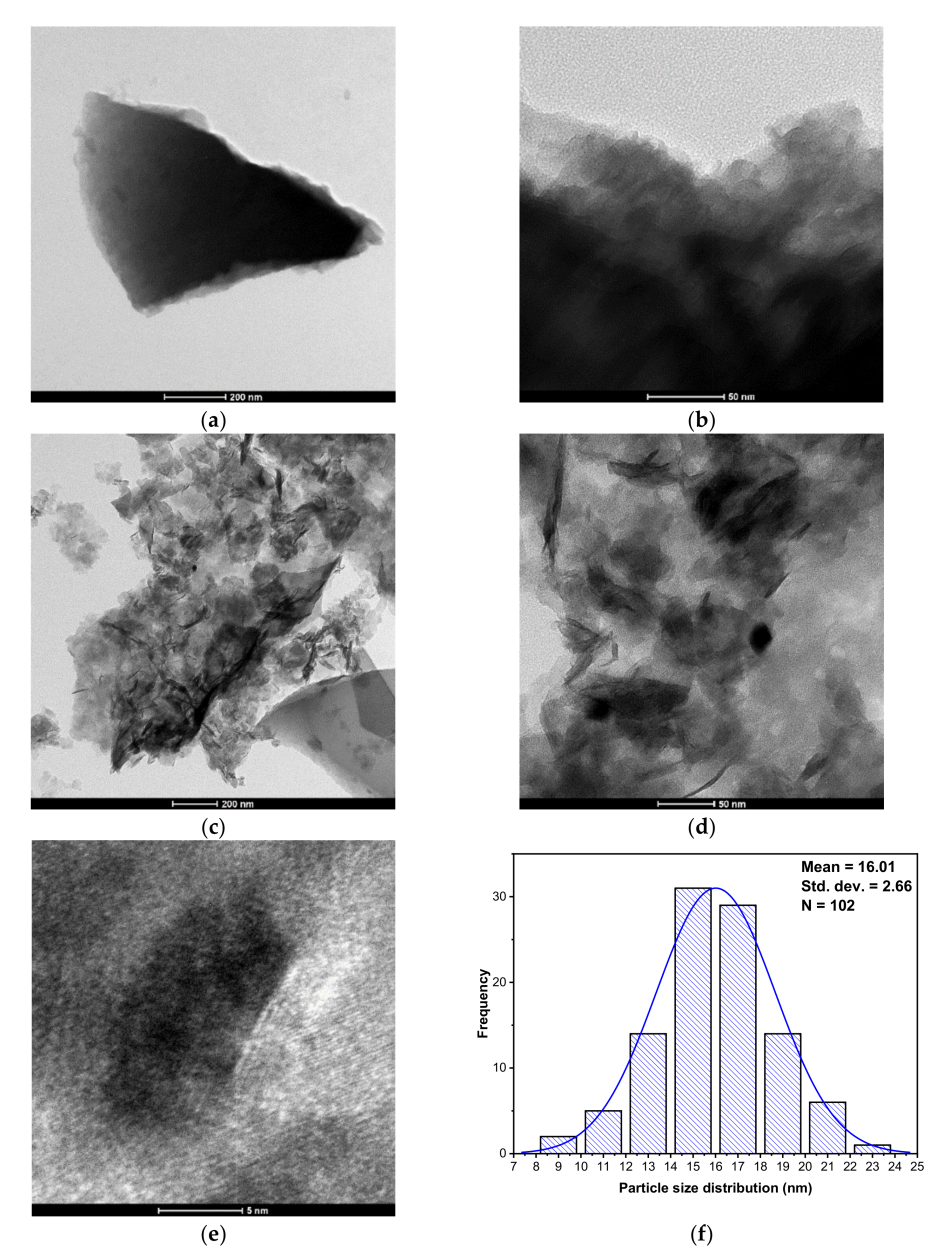
TEM images of the OSBC at two scales (**a**) 200 nm, (**b**) 50 nm and Co-OSBC at (**c**) 200 nm, (**d**) 50 nm, (**e**) 5 nm and (**f**) PSD results for the Co-OSBC sample.

TEM analysis was used for microstructural characterization of the synthesized nanoparticles on the surface of the biochar, Figure 6. The collected TEM images agreed with the SEM micrographs. The surface of the OSBC looks smooth (Figure 6a,b). On contrary, the surface of the Co-OSBC (Figure 6c–e) appears rough, with Co_3_O_4_ nanoparticles clearly identifiable on the surface. These nanoparticles had an average particle size of 16.01 ± 2.66 nm (Figure 6e). The synthesis of uniform sized Co_3_O_4_ nanoparticles on the surface of the OSBC is confirmed by a small particle size distribution (PSD) of 2.66 nm.

### 3.2. Full Factorial Design (FFD)

Assessment of the adsorption behavior of Co-OSBC was performed in a batch mode and following the scenario displayed in Table 3. Obtained responses were fitted to the polynomial model described by Equation (3).

(3) $$y=a_{0}+\overset{n}{\underset{i=1}{\sum }}a_{i}x_{i}+\overset{n}{\underset{i=1}{\sum }}\overset{n}{\underset{j=i+1}{\sum }}b_{ij}x_{i}x_{j}+\overset{n}{\underset{i=1}{\sum }}\overset{n}{\underset{j=i+1}{\sum }}\overset{n}{\underset{k=j+1}{\sum }}c_{ijk}x_{i}x_{j}x_{k}$$

where ***y*** denotes the theoretical response variable; %R or *q_e_* (mg/g), is the global mean, *x_i_, x_j_, x_k_* are the independent variables, and the coefficients: *a_i_*, *b_ij_*, *c_ijk_* symbolize the effects of single variable, interaction of two variables, and the interaction of three variables, respectively.

FFD is a screening design, which is commonly utilized when the number of variables is between 2–15. This design allows the estimation of the main effects of individual variables as well as their second-order interactions. For better response modelling, data transformation using Box-Cox response transformation tool was performed as displayed in Equation (4), where *λ* denotes the transformation factor [60].

(4) $$y^{\left(\lambda \right)}=\left\{\begin{array}{c} \frac{y^{\lambda }-1}{\lambda }\;\;\\ \log\left(y\right) \end{array}\begin{array}{c} \lambda \neq 0\;\;\;\\ \lambda =0\;\;\; \end{array}\right.$$

#### 3.2.1. Screening Phase

**Figure 7:**
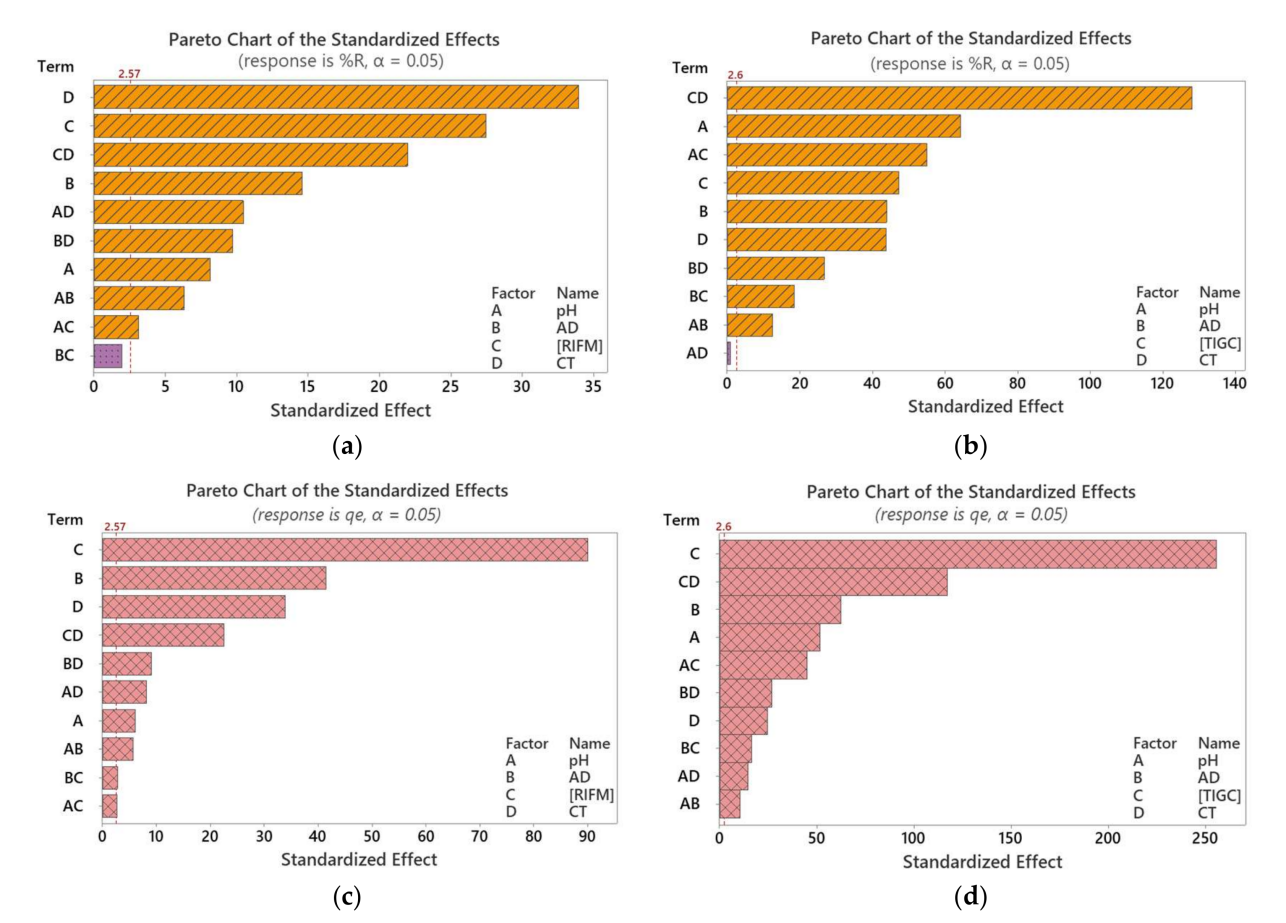
Pareto chart of the standardized effects: (**a**) %R (RIFM), (**b**) %R (TIGC), (**c**) *q_e_* (RIFM), and (**d**) *q_e_* (TIGC).

Pareto chart of the standardized effects was used to investigate the impact of each variable as well as their interactions on the assessed response(s). Figure 7—upper and lower panels show the Pareto charts in case %R and *q_e_* are the measured responses, respectively for both RIFM and TIGC. Taking %R as an example in case of RIFM, Figure 7a, CT (D), [RIFM] (C), and their two-way interaction (CD) were the most statistically significant variables. In contrary, in case of TIGC, Figure 7b shows that the interaction of [TIGC] × CT (CD), pH, and the interaction of pH × [TIGC] were the most influencing variables. Figure 7c,d show that [Antibiotic] was the most statistically significant variable influencing the adsorption of both drugs.

#### 3.2.2. Development of the Model Equations: Analysis of Variance (ANOVA)

Equations (5)–(8) describe the regression models obtained for both antibiotics. These shown models provide an illustration for the relation between the measured responses and the input variables. Different from Pareto charts, these equations describe the direction and the magnitude of the effect of each variable. Consequently, the entire impact of any factor could be merely assessed using these models. It is noteworthy to mention that Box-Cox response transformation was employed to obtain the optimized responses. The value of *λ* (transformation factor) was chosen to be ‘optimal’ for both responses in case of RIFM and as 0.10 in case of TIGC.

%R^λ^ _(RIFM)_ = −0.11804 + 0.006355 pH + 0.000372 AD − 0.000704 [RIFM] + 0.000498 CT − 0.000027 pH ×AD + 0.000015 pH × [RIFM] − 0.000041 pH × CT + 0.000001 AD × [RIFM] − 0.000002 AD × CT + 0.000004 [RIFM] × CT + 0.011780 Ct Pt. (5)

*q_e_*^λ^ _(RIFM)_ = 0.8499 + 0.03411 pH − 0.000201 AD + 0.004226 [RIFM] + 0.002760 CT − 0.000162 pH × AD + 0.000085 pH × [RIFM] − 0.000212 pH × CT − 0.000008 AD × [RIFM] − 0.000009 AD × CT + 0.000026 [RIFM] × CT + 0.15081 Ct Pt. (6)

%R^λ^ _(TIGC)_ = 0.8107 + 0.03905 pH + 0.000961 AD + 0.006259 [TIGC] + 0.000920 CT − 0.000050 pH × AD − 0.000383 pH × [TIGC] + 0.000091 pH × CT − 0.000009 AD × [TIGC] + 0.000007 AD × CT − 0.000034 [TIGC] × CT − 0.20789 Ct Pt. (7)

*q_e_^λ^*_(TIGC)_ = 0.58423 + 0.027174 pH − 0.000093 AD + 0.007043 [TIGC] + 0.000508 CT − 0.000055 pH × AD − 0.000246 pH × [TIGC] + 0.000073 pH × CT − 0.000009 AD × [TIGC] + 0.000005 AD × CT − 0.000024 [TIGC] × CT − 0.12739 Ct Pt. (8)

Model summaries are shown in Table 5. Each model was assessed using three parameters: the coefficient of determination (R^2^), R^2^–adjusted (R^2^–adj), and R^2^–predicted (R^2^–pred). The first two parameters were used to assess the model linearity. As shown in Table 5, values of both parameters were high indicating that these models were linear. On the other hand, the R^2^–pred was used to assess the model capability to predict the response for new trials, where the higher the value of R^2^–pred, the better the model capability. In the same itinerary, the difference between the experimental values and predicted values was assessed using the relative error (RE). Values of RE were relatively small indicating an agreement between experimental and predicted values.

Analysis of variance (ANOVA) test at 95.0 confidence level was used following the screening phase–Tables are not shown. A variable with a probability, *p*-value of ˃0.05 was considered as statistically insignificant. Findings of ANOVA agreed with the conclusions derived from the regression equations and Pareto charts.

#### 3.2.3. Optimization Phase

**Figure 8:**
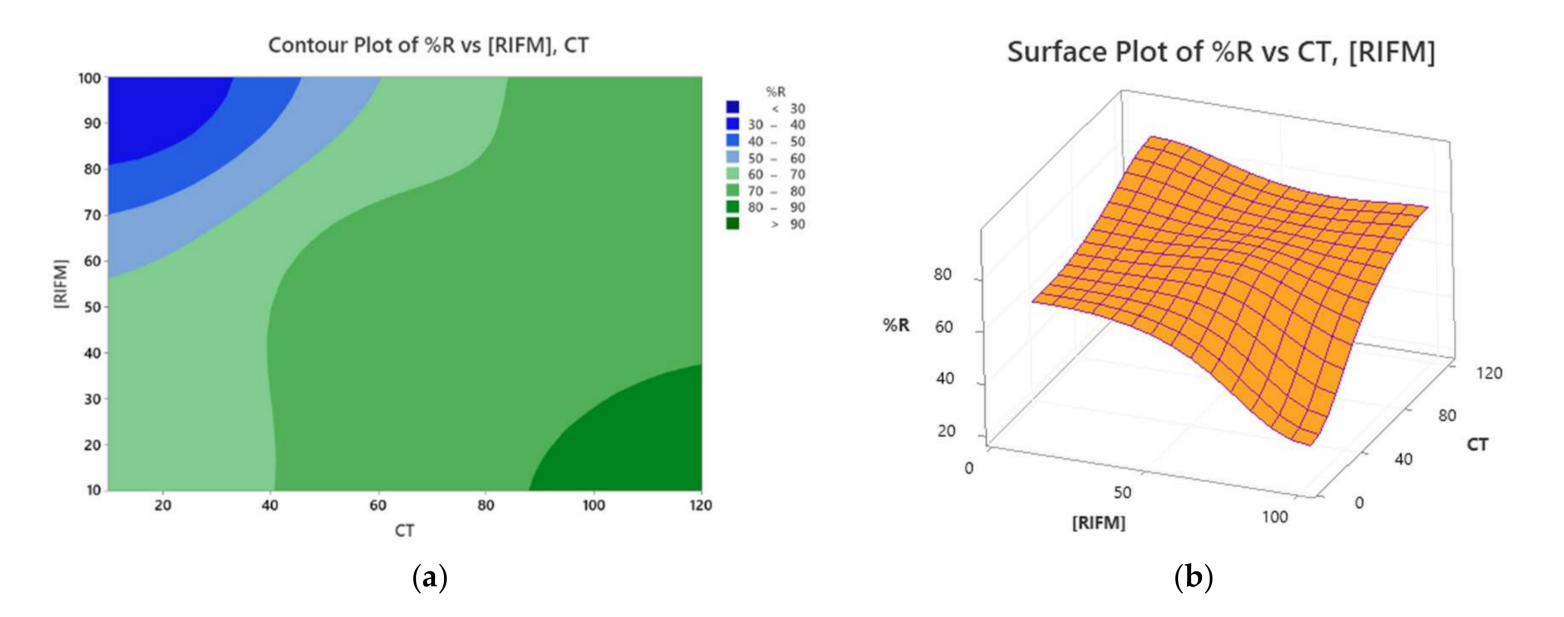
(**a**) Two-dimensional contour plot, and (**b**) three-dimensional surface plot between CT (min) and [RIFM], ppm for the removal of RIFM.

Following the screening of the variables affecting the removal of RIFM and TIGC and ANOVA, an optimization phase was run using a variety of tools. Among these tools, the contour and surface plots were drawn. Contour plots are two dimensional plots used to describe the relation between two variables and the response variable using the contour lines. Surface plots, however, relate two variables and the response surface using the three-dimensional format. Sample contour and surface plots (%R is the response measured) are shown in Figure 8 in case of RIFM. As shown in Figure 8a, the darkest green zone is the region where the combination of both variables could achieve the highest removal of RIFM. Figure 8b is a surface plot for the same two independent variables in a and the response. The high edge represents the points of maximum response.

Another tool, optimization plots—Figures are not shown, were also used to find the values of optimum conditions that could maximize the measured response(s). Desirability value (*d*) was used as an indicator for the optimum factorial blend, where the higher the value of the desirability function, the better is the described blend and the higher the response. The optimum conditions together with the maximum desirability achieved are shown in Table 5.

The findings of the design optimization further support the conclusions obtained from the characterization techniques. The pH, and as per the optimization data is not the most statistically significant variable in case of RIFM, an issue that suggests that removal of RIFM might not occur via chemisorption. In case of TIGC, however, pH was the second most statistically significant variable. These assumptions will be further confirmed using the equilibrium studies.

### 3.3. Adsorption Isotherms and Kinetic Studies

In general, adsorption performance is determined by the functionalities present on the adsorbent’s surface as well as its surface area. Different types of interactions could be proposed for the interaction of RIFM and TIGC with Co-OSBC based on the findings of the characterization data as well as the FFD design output.

#### 3.3.1. Adsorption Isotherms

Adsorption isotherms may be used to assess the amount of adsorbate accumulation on the adsorbent’s surface as well as the type of adsorbent-adsorbate interaction. The adsorption of RIFM and TIGC onto Co-OSBC at a constant temperature was investigated using four equilibrium isotherms: Langmuir, Freundlich, Temkin, and Dubinin–Radushkevich (D–R) [61,62,63,64].

**Figure 9:**
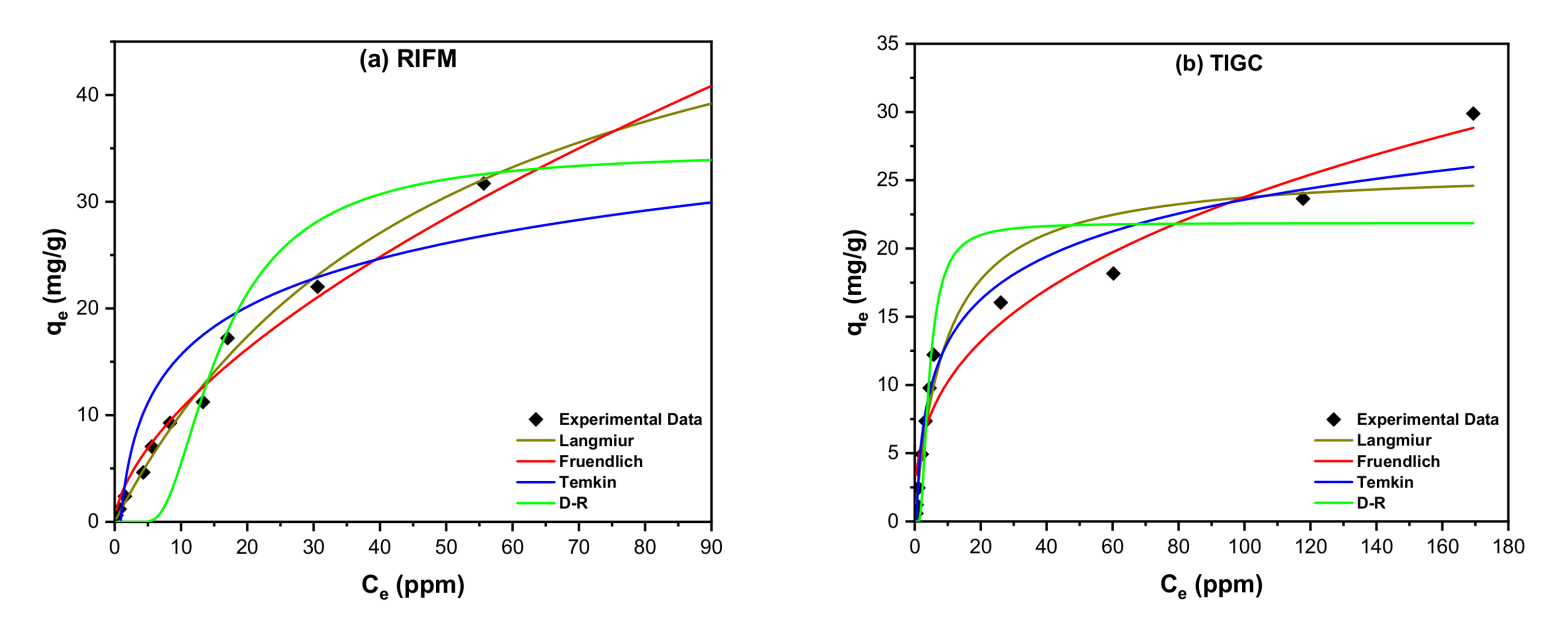
Adsorption isotherms of Co-OSBC adsorbent with both RIFM (**a**) and TIGC (**b**) including Langmuir, Freundlich, Temkin, and Dubinin–Radushkevich (D–R) isotherms.

The Langmuir isotherm often implies one of three primary hypotheses: (I) the adsorption sites present on the adsorbent surface have identical adsorption energy, (II) the adsorbate molecule occupied each on one site on the surface of the adsorbent, and there is no interaction between the molecules of the adsorbate (III) the adsorption is mainly localized on the surface of the adsorbent. It is represented by Equation (9) and Figure 9a,b for the RIFM and TIGC, respectively.

(9) $$q_{e}=\frac{q_{m}\;K_{L}\;C_{e}}{1+K_{L}\;C_{e}}$$

where *q_m_* represents the maximal adsorption capacity and *K_L_* represents the Langmuir equilibrium coefficient. Furthermore, the Langmuir model may be expressed in the dimensionless form illustrated by Equation (10):

(10) $$R_{L}=\frac{1}{1+K_{L}\;C_{0}}$$

where *R_L_* and *C*_0_ (ppm) denote the separation factor and initial concentration of RIFM and TIGC, respectively. The *R_L_* value can assess adsorption desirability; thus, if *R_L_* is 1, the adsorption process is deemed unfavorable; if *R_L_* = 1, the adsorption occurs linearly; but if the value is between 0–1, the adsorption is considered favorable and can occur spontaneously. If the *R_L_* value is 0, the adsorption is irreversible. The determined *R_L_* value for the adsorption of both RIFM and TIGC onto Co-OSBC was found to be less than 1, showing that the adsorption process was spontaneous. Furthermore, the adsorption of the two drugs became irreversible at higher concentrations of both drugs with maximum adsorption capacity (*q_max_*) = 61.10 and 25.94 mg/g for RIFM and TIGC, respectively. The obtained data indicates that Co-OSBC, as an adsorbent, has a greater adsorptive capability for RIFM than the TIGC drug.

The Freundlich isotherm is a totally empirical technique for describing the energy of a heterogeneous surface, and it is represented by Equation (11):

(11) $$q_{e}=\;K_{F}C_{e}^{\frac{1}{n}}$$

where *C_e_* is the equilibrium concentration of RIFM and TIGC (ppm), *q_e_* is the amount of drug adsorbed/unit mass (mg·g^−1^), *K_F_* (mol·g^−1^)(L·mol^−1^) and 1/*n* are the Freundlich coefficients that express the adsorbent capacity and change in adsorption intensity, as well as the deviation from linearity. The obtained data shown in Figure 9a,b for the RIFM and TIGC, respectively, and their values are listed in Table 6. The Freundlich isotherm data fit well, with R^2^ values of 0.9894 and 0.9481 for both RIFM and TIGC, respectively, which are higher than the R^2^ values obtained for the Langmuir isotherm (R^2^ = 0.9748 for RIFM and 0.9299 for TIGC), implying that Freundlich isotherms can be used to describe the adsorption of TIGC and RIFM onto the as-prepared adsorbent Co-OSBC. Table 6 reveals that the RIFM has a 1/*n* = 0.61, *n* = 1.628 while the TIGC has a 1/*n* = 0.37, *n* = 2.727. As a result, the adsorption potential (A = *n*RT) for RIFM is 5.45 kJ and 9.13 kJ for TIGC, meaning that any RIFM molecule with a potential energy of 5.45 kJ may be adsorbed onto the surface of Co-OSBC, and the adsorption is favorable and irreversible.

Figure 9a,b depicts the Temkin isotherm, which describes the interaction between the adsorbate and the adsorbent; the heat of adsorption of adsorbed molecules in a layer decreases linearly with the adsorbent–adsorbate interactions. According to the results in Table 6, RIFM has a sorption energy of 381.28 J/mol, and the TIGC has a sorption energy of 544.71 J/mol. These findings suggest that RIFM and TIGC adsorb favorably onto the as-prepared adsorbent and confirm the data obtained from the Langmuir and Freundlich isotherms.

Finally, the D–R equilibrium isotherm at room temperature was investigated, as shown in Figure 9a,b and Table 6. The acquired results for both drugs reveal that the RIFM sorption energy is 2.746 kJ/mol and 9.391 kJ/mol for the TIGC, indicating that the RIFM adsorption onto Co-OSBC is physisorption with sorption energy less than 7 kJ/mol. The adsorption of TIGC, on the other hand, is chemisorption since the adsorption energy is greater than 7 kJ/mol, implying that the adsorption of TIGC onto the investigated adsorbent is dependent primarily on the presence of functional groups on the adsorbent’s surface. However, RIFM adsorption is mainly determined by the surface area of Co-OSBC. Furthermore, the maximum adsorption capacity of TIGC on Co-OSBC is 21.87 mg/g, which is similar to the maximum adsorption capacity of Langmuir.

#### 3.3.2. Kinetic Studies

**Figure 10:**
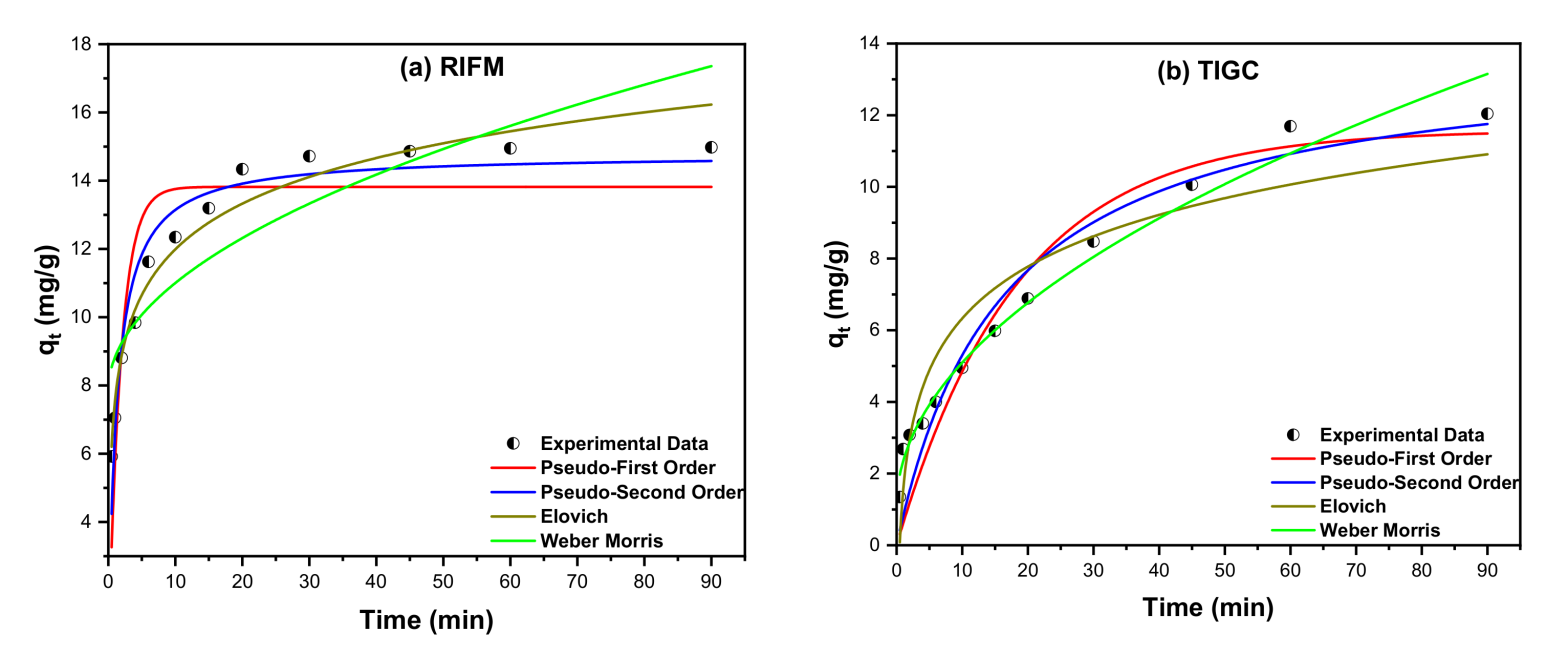
Kinetic models for the adsorption of RIFM (**a**) and TIGC (**b**) onto Co-OSBC.

The adsorption process of both RIFM and TIGC onto the adsorbent Co-OSBC was studied using four kinetic models: pseudo–first order (PFO), pseudo–second order (PSO), Elovich, and Weber–Morris (WM) [65,66,67]. Figure 10a,b illustrate the relationship between *q_t_* (mg/g) vs time (min) for the adsorption of RIFM and TIGC onto Co-OSBC, respectively. Table 7 displays the calculated parameters for the four models. The acquired results suggest that the R^2^ value for the PSO model is greater for the adsorption of both drugs onto Co-OSBC (0.9356 for RIFM and 0.9346 for TIGC). These findings show that the rate of the adsorption process is affected by both the drug and the adsorbent, and the adsorption reaction could be expressed as follows, Equation (12):

(12) $$\mathrm{RIFM}\;\&\;\mathrm{TIGC}+\mathrm{Co}–\mathrm{OSBC}\;\left(\overset{k}{\to }\right)\;\left\{\mathrm{RIFM}–\mathrm{Co}–\mathrm{OSBC}\right\}or\;\left\{\mathrm{TIGC}–\mathrm{Co}–\mathrm{OSBC}\right\}$$

The Elovich model, on the other hand, reveals large initial adsorption for RIFM (96.22 mg·g^−1^·min^−1^) and lesser initial adsorption for TIGC (4.34 mg·g^−1^·min^−1^). Finally, the R^2^ value of the Weber–Morris (WM) was too low for RIFM (0.7582) compared to the other models, although it is higher (0.9802) for TIGC; hence, this model may be utilized to represent TIGC adsorption onto Co-OSBC.

## 4. Conclusions

Based on the data presented in this work, an efficient and cost-effective nanosorbent could be obtained by loading Co_3_O_4_ nanoparticles onto the olive stones biochar (Co-OSBC). The adsorption of two PhACs, namely rifampicin (RIFM) and tigecycline (TIGC) was successfully achieved using the developed nanosorbent. The developed nanosorbent (Co-OSBC) showed a higher removal efficiency (%R) and adsorption capacity (*q_e_*) towards RIFM compared to TIGC. A %R of 95.18% could be achieved for RIFM, compared to 75.48% for TIGC. FT-IR analysis of Co-OSBC before and after adsorption of RIFM showed slight shifts in the position of some functional groups confirming the adsorption of RIFM onto the nanosorbent surface. Raman analysis confirmed the presence of Co_3_O_4_ nanoparticles on the surface of the OSBC. BET analysis and SEM micrographs showed the presence of both meso- and macropores on the surfaces of the Co-OSBC, and the later showed a higher surface area (39.85 m^2^/g) and pore volume (0.168 cm^3^/g) compared to the pristine OSBC sample. Moreover, A multivariate approach–two-level full factorial design (2*^k^*-FFD) was utilized to optimize the dependent responses (%R and *q_e_*). Our goal was to achieve the highest removal of both RIFM and TIGC and the highest adsorption capacity via the lowest consumption of resources and chemicals. Design analysis showed that the optimum conditions for the highest %R of RIFM for example were pH = 5.0 ± 0.2, AD = 150 mg/13 mL, [RIFM] = 10 ppm and CT = 120 min. Nonlinear fittings equilibrium studies showed that the adsorption of both RIFM and TIGC was favorable with a maximum adsorption capacity (*q_max_*) of 61.10 mg/g in the case of RIFM compared to 25.94 mg/g for TIGC. D–R equilibrium isotherm revealed that the adsorption of RIFM onto Co-OSBC was physisorption while TIGC was chemically adsorbed. The adsorption kinetics indicated that the adsorption of RIFM and TIGC were perfectly fit the PSO model.

## Figures and Tables

**Table 1:** Adsorptive removal of antibiotics from wastewater using BC-based sorbents loaded with nanoparticles.

Feedstock	Nanosorbent	Antibiotic(s)	Method of Nanoparticles Preparation	Parameters Optimization	Surface Area (m^2^/g)	*q_e_* (mg/g)	%R	Ref
Olive Stones	Cobalt Oxide (Co_3_O_4_)-Olive Stone Biochar (Co-OSBC)	Rifampicin (RIFM) Tigecycline (TIGC)	Microemulsion Method	Full Factorial Design (FFD)	39.85	61.1025.94	95.18 75.48	Current Study
Camphor Leaf	ZnO Nanoparticle Modified Magnetic Biochar	Ciprofloxacin	Ultrasound Assisted Wet-Chemical Method	Univariate Analysis	950	449.40 *	** NS	[41]
*Banana Pseudostem* (BP) Fibers	CoFe_2_O_4_-Modified Biochar (BP350 and BP650)	Amoxicillin	Co-Precipitation	Univariate Analysis	BP350:100.9 BP650:190.5	99.99	** NS	[42]
*Camellia oleifera* Shells	Cobalt-Gadolinium Modified Biochar	Ciprofloxacin Tetracycline	Mixing of Co(II) and Gd(III) with the Biochar, Shaking, Drying then Pyrolysis	Univariate Analysis	370.3737	44.44 119.05	System-Dependent	[43]
Sawdust	Modified Biochar Supported Co/Fe Nanoparticles (Co/Fe/MB)	Cefotaxime	Liquid Phase Reduction Method under Anaerobic Conditions	Univariate Analysis	262	30.07	99.23	[44]
Olive Kernel	Magnetic Adsorbent Prepared from Olive Kernel	Amoxicillin	Ultrasonic Process	Univariate Analysis	2188.0	238.1	** NS	[45]
Vinasse Wastes	Ferromanganese Modified Biochar (Fe/Mn-BC)	Levofloxacin	Co-Precipitation	Univariate Analysis	93.4	181	** NS	[46]
Spent Coffee Grounds	Spent Coffee Grounds Biochar Impregnated with Titanium Oxide (TiO_2_)	Balofloxacin	Hydrothermal Method with Modifications	Plackett-Burman Design	50.54	196.73	91.78	[47]

* Reported unit is mg/L, ** NS: Not stated.

**Table 2:** Investigated variables and their upper, mid-, and lower levels.

Factors and Units	−1	0	+1
pH (A, pH Unit)	5.0	7.0	9.0
Sorbent Dosage (AD, B, mg/13 mL)	50.0	100.0	150.0
Initial Antibiotic Concentration ([Antibiotic], C, ppm)	10.0	55.0	100.0
Contact Time (CT, D, min)	10.0	65.0	120.0
Response Variables	Percentage Removal (%R)		
	Adsorption Capacity (*q_e_*_,_ mg/g)		

**Table 3:** Design matrix. Experimental values for %R and *q_e_* are shown.

Run Number	Block	Variables				RIFM		TIGC	
		pH	AD	[Antibiotic]	CT	%R	*q_e_*	%R	*q_e_*
01	2	9 (+)	150 (+)	100 (+)	10 (−)	38.80	3.35	26.24	2.27
02	2	5 (−)	50 (−)	10 (−)	10 (−)	42.34	1.07	2.64	0.07
03	2	9 (+)	50 (−)	10 (−)	120 (+)	82.59	2.15	32.37	0.84
04	2	7 (0)	100 (0)	55 (0)	65 (0)	76.00	5.43	2.96	0.21
05	2	5 (−)	150 (+)	100 (+)	120 (+)	87.08	7.55	14.47	1.25
06	1	9 (+)	50 (−)	100 (+)	120 (+)	69.98	18.20	11.48	2.98
07	1	9 (+)	150 (+)	10 (−)	10 (−)	83.93	0.73	10.66	0.09
08	1	5 (−)	50 (−)	100 (+)	10 (−)	19.86	5.10	29.05	7.39
09	1	5 (−)	150 (+)	10 (−)	120 (+)	95.18	0.82	27.89	0.24
10	1	7 (0)	100 (0)	55 (0)	65 (0)	73.43	5.25	2.85	0.20
11	4	7 (0)	100 (0)	55 (0)	65 (0)	73.90	5.28	3.86	0.28
12	4	5 (−)	150 (+)	100 (+)	10 (−)	32.74	2.84	39.08	3.39
13	4	9 (+)	150 (+)	100 (+)	120 (+)	83.84	7.27	19.80	1.72
14	4	9 (+)	50 (−)	10 (−)	10 (−)	71.04	1.85	10.32	0.27
15	4	5 (−)	50 (−)	10 (−)	120 (+)	91.45	2.38	11.46	0.30
16	3	5 (−)	150 (+)	10 (−)	10 (−)	76.52	0.66	3.55	0.03
17	3	9 (+)	50 (−)	100 (+)	10 (−)	29.38	7.64	26.31	6.84
18	3	7 (0)	100 (0)	55 (0)	65 (0)	74.20	5.31	2.35	0.17
19	3	9 (+)	150 (+)	10 (−)	120 (+)	77.59	0.67	75.46	0.65
20	3	5 (−)	50 (−)	100 (+)	120 (+)	64.19	16.69	7.31	1.90

**Table 4:** BET analysis of the as-prepared sorbents; OSBC and Co-OSBC.

Parameters	OSBC	Co-OSBC
Langmuir Surface Area (m^2^/g)	22.20	39.85
Total Pore Volume (cm^3^/g)	0.086	0.168
Average Pore Radius (Å)	86.1	93.0

**Table 5:** Parameters used to assess the regression equations, Equations (5)–(8) and the optimum conditions.

Contaminant	Response	R^2^%	R^2^–adj%	R^2^–pred%	Optimum Conditions and Desirability (*d*) Values
RIFM	%R	99.84	99.39	98.28	pH = 5.0, AD = 150 mg, [RIFM] = 10 ppm, CT = 120 min, (*d* = 1.0000, %R = 100%)
	*q_e_*	99.96	99.85	99.45	pH = 9.0, AD = 50 mg, [RIFM] = 100 ppm, CT = 120 min, (*d* = 1.0000, *q_e_* = 18.30 mg/g)
TIGC	%R	99.82	99.31	96.06	pH = 9.0, AD = 150 mg, [TIGC] = 10 ppm, CT = 120 min, (*d* = 1.0000, %R = 82.37%)
	*q_e_*	99.99	99.97	99.87	pH = 9.0, AD = 50 mg, [TIGC] = 100 ppm, CT = 10 min, (*d* = 1.0000, *q_e_* = 7.83 mg/g)

**Table 6:** Nonlinear equations of Langmuir, Freundlich, Temkin and Dubinin–Radushkevich (D–R) isotherms, besides their parameters for the adsorption of both RIFM and TIGC onto Co-OSBC adsorbent.

Isotherm	Equations (Nonlinear Forms)	Parameters	Value	
			RIFM	TIGC
**Langmuir**	$q_{e}=\frac{q_{m}\;K_{L}\;C_{e}}{1+K_{L}\;C_{e}}$	*q_m_* (mg/g)	61.10	25.94
		*K_L_* (L·mol^−1^)	0.019	0.108
		R^2^	0.9748	0.9299
**Freundlich**	$q_{e}=K_{F}C_{e}^{\frac{1}{n}}$	1/*n*	0.61	0.37
		*K_F_* (mol/g) (L/mol)^1/n^	2.57	4.39
		R^2^	0.9894	0.9481
**Temkin**	$q_{e}=\frac{RT}{b_{T}}\;\ln\left(A_{T}\;C_{e}\right)$	*b_T_* (J/mol)	381.28	544.71
		*A_T_* (L/mol)	1.112	1.783
		R^2^	0.8091	0.9629
**D–R**	$q_{e}=\;q_{s\;}$$\;\exp\;(-\beta \cdot \epsilon^{2}$)	β	6.63 × 10^−8^	5.67 × 10^−9^
	$\epsilon =RT\left(1+\frac{1}{C_{e}}\right)$	*E* (kJ/mol)	2.746	9.391
		*q_m_* (mg/g)	34.76	21.87
	E=12β	R^2^	0.8839	0.8568

*q_e_*: amount of adsorbate in the adsorbent at equilibrium, K_L_: Langmuir isotherm constant, *q_max_*_:_ maximum monolayer coverage capacities, *K_F_*: Freundlich adsorption constant, *C_e_* equilibrium concentration, *q_s_*: theoretical isotherm saturation capacity, *A_T_*: Temkin isotherm equilibrium binding constant, R: universal gas constant (8.314 J/mol K), *T* is the temperature (K), *b_T_*: Temkin isotherm constant.

**Table 7:** Data for the kinetic studies corresponding to the data shown in Figure 10.

Models	Parameter	Value	
		RIFM	TIGC
$\mathrm{Pseudo}-\mathrm{first}\;\mathrm{order}\;(\mathrm{PFO})\;\frac{dq_{t}}{dt}$*= k*_1_(*q_e−_q_t_*)	K_1_ (min^−1^)	0.539	0.0543
	*q_e_* (mg/g)	13.82	11.58
	R^2^	0.7907	0.9085
$\mathrm{Pseudo}-\sec\mathrm{ond}\;\mathrm{order}\;(\mathrm{PSO})\;\frac{dq_{t}}{dt}$*= k*_2_(*q_e−_q_t_*)^2^	K_2_ *(*g·mg^−1^·min^−1^*)*	0.054	0.004
	*q_e_* (mg/g)	14.78	13.86
	R^2^	0.9356	0.9346
$\mathrm{Elovich}\;\mathrm{model}\;q_{t}\;\frac{1}{\beta }\times ln\left(1+\alpha \beta t\right)$	*α*	96.225	4.339
	*β*	0.518	0.4796
	R^2^	0.9624	0.9033
$\mathrm{Weber}-\mathrm{Morris}\;\mathrm{model}\;(\mathrm{WM})\;q_{t}=K_{I}t^{0.5}+C$	*K_I_*	1.004	1.273
	*C*	7.826	1.071
	R^2^	0.7582	0.9802

where *K*_2_ is rate constant (g·mg^−1^·min^−1^) and *q_t_* is adsorbed quantity at time t; while α and β are initial sorption concentration rate (mg·g^−1^·min^−1^), and desorption constant (g/mg), *K_I_* is intraparticle diffusion rate constant (mg·g^−1^·min^−0.5^), and *C* is boundary thickness effect.

## Data Availability

The data presented in this study are available within this article. Further inquiries could be directed to the authors.
